# A Transfer Learning Algorithm to Reduce Brain-Computer Interface Calibration Time for Long-Term Users

**DOI:** 10.3389/fnrgo.2022.837307

**Published:** 2022-04-21

**Authors:** Joshua Giles, Kai Keng Ang, Kok Soon Phua, Mahnaz Arvaneh

**Affiliations:** ^1^Department of Automatic Control and Systems Engineering, The University of Sheffield, Sheffield, United Kingdom; ^2^Institute for Infocomm Research, Agency for Science, Technology and Research, (A*STAR) Singapore, Singapore; ^3^School of Computer Science and Engineering, Nanyang Technological University, Singapore, Singapore

**Keywords:** session to session transfer learning, long-term BCI users, reducing calibration time, EEG, motor imagery, Brain-Computer Interfaces

## Abstract

Current motor imagery-based brain-computer interface (BCI) systems require a long calibration time at the beginning of each session before they can be used with adequate levels of classification accuracy. In particular, this issue can be a significant burden for long term BCI users. This article proposes a novel transfer learning algorithm, called r-KLwDSA, to reduce the BCI calibration time for long-term users. The proposed r-KLwDSA algorithm aligns the user's EEG data collected in previous sessions to the few EEG trials collected in the current session, using a novel linear alignment method. Thereafter, the aligned EEG trials from the previous sessions and the few EEG trials from the current sessions are fused through a weighting mechanism before they are used for calibrating the BCI model. To validate the proposed algorithm, a large dataset containing the EEG data from 11 stroke patients, each performing 18 BCI sessions, was used. The proposed framework demonstrated a significant improvement in the classification accuracy, of over 4% compared to the session-specific algorithm, when there were as few as two trials per class available from the current session. The proposed algorithm was particularly successful in improving the BCI accuracy of the sessions that had initial session-specific accuracy below 60%, with an average improvement of around 10% in the accuracy, leading to more stroke patients having meaningful BCI rehabilitation.

## 1. Introduction

Brain-computer interface (BCI) allows a person to communicate with a machine directly through brain signals alone (Berger et al., 2008; Ang and Guan, 2017). Electroencephalogram (EEG)-based BCIs, in particular, are very popular as the brain signals can be recorded non-invasively with a high temporal resolution while being inexpensive (Berger et al., 2008; Vidaurre et al., 2011). From the EEG signals, a range of features associated with different mental states and cognitive functions can be identified and then used to control a BCI (Berger et al., 2008). This includes BCI systems based on detecting P300, motor imagery, the user's mental workload, and emotions. Motor imagery in particular can be very useful in healthcare, with studies linking the use of motor imagery-based BCIs with neuro-rehabilitation in stroke patients (Buch et al., 2008; Ang et al., 2010). A motor imagery-based BCI provides an active rehabilitation by coupling motor relevant brain activities with the physical movements of the impaired limb (Ang et al., 2010; Ang and Guan, 2015).

Typically, motor imagery-based BCIs require calibration before each session of use, where new labeled EEG data is collected to train the BCI model. This calibration session is fatiguing for the user (Costa et al., 2018). CSP-based BCI commonly require 15–30 min of calibration, collecting 40, 60 or even 80 trials per class to train the BCI (Lotte, 2015; Proesmans, 2016). This issue is a significant barrier for the use of BCI by stroke patients as they require regular BCI sessions for rehabilitation (O'Dell et al., 2009). The time spent on calibrating the system in each of these sessions reduces the time available for the patient to receive the actual rehabilitation.

To reduce the BCI calibration time transfer learning can be used (Azab et al., 2018). Transfer learning is a commonly employed technique in systems engineering when only a limited amount of data is available to train the model. Transfer learning compensates for the limited amount of labeled data available by extracting relevant information from other sources or domains to improve the classification model (Azab et al., 2018). However, transfer learning in BCI is not a trivial task due to the non-stationary nature of brain signals. The properties of EEG signals often change considerably from session to session (Dikanev et al., 2005).

To reduce the affects of the non-stationarities, a range of approaches have been explored and embedded in transfer learning algorithms proposed for BCI (Hossain et al., 2016; Peng et al., 2020). For example, some transfer learning algorithms applied alignment of the EEG distributions between the source and target sessions (Sugiyama et al., 2008; Dalhoumi et al., 2015; Raza et al., 2016; He and Wu, 2018) or weighted the source sessions according to their similarities with the target session (Azab et al., 2019b). However these research studies focused on inter-subject transfer learning, evaluating the proposed solutions on datasets with only one or two sessions of data available for each subject. There is a research bias within BCI, with the majority of studies focusing on datasets where only one or two sessions are recorded from each subject (Perdikis and Millan, 2020). There is a relatively small amount of literature focused on long-term users and inter-session transfer learning. One of the main causes of inter-subject variations in EEG signals is the varying brain morphology across the users. This issue is particularly significant for stroke users whose brain is altered by lesions that vary in size and location from user to user. Inter-session transfer learning is not affected by this issue with the majority of the non-stationarities coming from other causes.

Despite the limited number of studies on long-term BCI users, the potential benefits of inter-session transfer learning to reduce the calibration period is clear. Arvaneh et al. found that when 11 previous sessions, with 60 trials of each class in each session, were combined in the form of a “naive transfer learning”, potentially invariant BCI features could be identified (Arvaneh et al., 2012). These sessions were collected over a period of 1 month and showed the potential of inter-session transfer learning to reduce the need for a calibration session. However, this approach is very limited. A lot of data is required before these so-called invariant features can be extracted, while some users still continue to perform better with the BCI model trained only using the data collected from the new session, called the “session-specific model”.

One of the key sources of literature on inter-session transfer learning for long-term BCI users is from the teams competing in the Cybathlon BCI event (Perdikis et al., 2018). The BCI race at the Cybathlon competitions has been held every 4 years where teams with tetraplegic pilots competed to control an avatar through a race track using BCI (Novak et al., 2018). This required the teams to develop BCIs that could detect three different mental commands and to train a pilot to use the system. The user training period ranged from a month to over a year for some teams, allowing an in-depth exploration of BCI for long-term use and the potential of inter-session transfer learning.

The team led by Hehenberger et al. utilized inter-session transfer learning and intra-session adaptation for their BCI model developed for the Cybathlon competition race (Hehenberger et al., 2021). This team worked with their pilot for 14 months collecting 26 sessions, each containing 120 EEG trials. Using the collected dataset, in an offline analysis, the authors highlighted the benefits of inter-session transfer learning over session-specific BCIs. They combined the data from the past five sessions and the new data to train the BCI model in the form of an inter-session naive transfer learning. Although it was successful to some extent, no optimization was performed when combining the new and previous data to reduce the inter-session non-stationarities. Another Cybathlon team led by Benaroch et al. explored using both inter-session alignment and intra-session adaptation for their BCI model (Benaroch et al., 2021). Over a period of 3 months this team collected 20 sessions, with the length of the sessions varying between an hour and 2 h. To reduce the calibration time and reduce inter-session variability, they applied an alignment method projecting the Riemannian mean spatial covariance matrix from each session to a common reference point (i.e., the identity matrix). This alignment proved effective at reducing the non-stationarities and improving the inter-session transfer learning. However, even with this alignment still some of the source sessions were detrimental to the BCI model. Other researchers suggested that the use of selective transfer learning would reduce the effects of detrimental source sessions by weighting the source sessions based on their similarities with the target session (Wei et al., 2016).

Recently, Cao et al. has explored weighting the source sessions to improve the inter-session transfer learning in long-term BCI users performing BCI-based stroke rehabilitation (Cao et al., 2021). For this purpose, they utilized a previously proposed inter-subject transfer learning algorithm (Azab et al., 2019b). The proposed transfer learning algorithm added a regularization parameter to the objective function of the BCI classifier, aiming at minimizing the dissimilarity between the classification parameters of the new session and the past sessions while maximizing the two class separations. Importantly, the proposed algorithm gave different weights to different source classifiers based on the similarity between their features and the features of the target session. Cao et al. validated the utilized inter-session transfer learning algorithm on a BCI dataset from seven stroke patients. The dataset consists of 12 BCI sessions per stroke patient, each session having 180 trials performed in a randomized order. The proposed inter-session transfer learning algorithm significantly increased the classification accuracy of stroke subjects encountering BCI deficiency. However the improvement in BCI accuracy was not statistically significant for all users. Many of the stroke participants performed better when relying on the standard naive transfer learning; with all source sessions having the same weight.

This article focuses on inter-session transfer learning to reduce the required calibration time for stroke patients who use BCI for rehabilitation. The proposed algorithm reduces this calibration time by combining previously recorded data from the same user with a limited number of data recorded from the current session, reducing the need for an extended calibration session. The proposed algorithm, called r-KLwDSA, consists of three steps to make effective use of the inter-session source sessions. Initially, the algorithm uses linear alignment to reduce non-stationarity between the current target session and the previous source sessions. The source sessions are then weighted to minimize the effects of any detrimental source data. Finally, the algorithm utilizes regularization to incorporate the target data and the weighted aligned source data into the BCI model.

The proposed r-KLwDSA algorithm is evaluated using EEG signals collected from 11 stroke patients over a period of 6 weeks. To simulate the real world scenario of long-term BCI use for stroke rehabilitation, the data will be evaluated chronologically, only using previously collected sessions for transfer learning. As such when evaluating the first target session only the screening session will be available for inter-session transfer learning and when evaluating session two both the screening session and session one will be used for transfer learning. This article will compare the effects of the weighting and alignment separately as well as the combined effect on classification performance. Furthermore, the performance of the proposed r-KLwDSA algorithm will also be compared with the performance of the session-specific BCI model trained with only the available trials from the current target session and the naive transfer learning model trained with only the previous source sessions without any alignment.

## 2. Methodology

In this article, we assume that EEG trials of *J* sessions, previously recorded from our current user, are available. These sessions are called source sessions. The *j*^*th*^ source session, $\hat{{\text{S}}_{\text{j}}}$, can be represented as $\hat{{\text{S}}_{\text{j}}}\;=\;{\left({\hat{X}}_{j}^{i},{\hat{y}}_{j}^{i}\right)}_{i=1}^{{\hat{m}}_{j}}$, where ${\hat{\text{X}}}_{j}^{i}$ ∈ $\hat{{\text{X}}_{j}}$ ⊂*R*^*ch*×*t*^ represents the *i*^*th*^ EEG trial from ${\hat{m}}_{j}$ total EEG trials available in $\hat{{\text{S}}_{\text{j}}}$, and ${ŷ}_{j}^{i}\in \hat{{\text{y}}_{j}}\subset \mathbb{R}$ represents the corresponding class label. Moreover, *ch* and *t*, respectively, denote the number of channels and the number of time samples recorded in each EEG trial.

Similarly, in this article, we have access to a small number of EEG trials from a new session, collected in a short calibration session from the same user. This session, referred to as the target session, is presented as $\text{S }\;=\;\;{\left(X^{\text{i}},{\text{y}}^{\text{i}}\right)}_{\text{i=1}}^{\text{m}}$, where **X**^*i*^ ∈ **X** ⊂ *R*^*ch*×*t*^ is the *i*^*th*^ recorded trial and *y*^*i*^ ∈ **y** ⊂ ℝ represents its corresponding class label. Moreover, *m* refers to the total number of trials in the target session.

**Figure 1:**
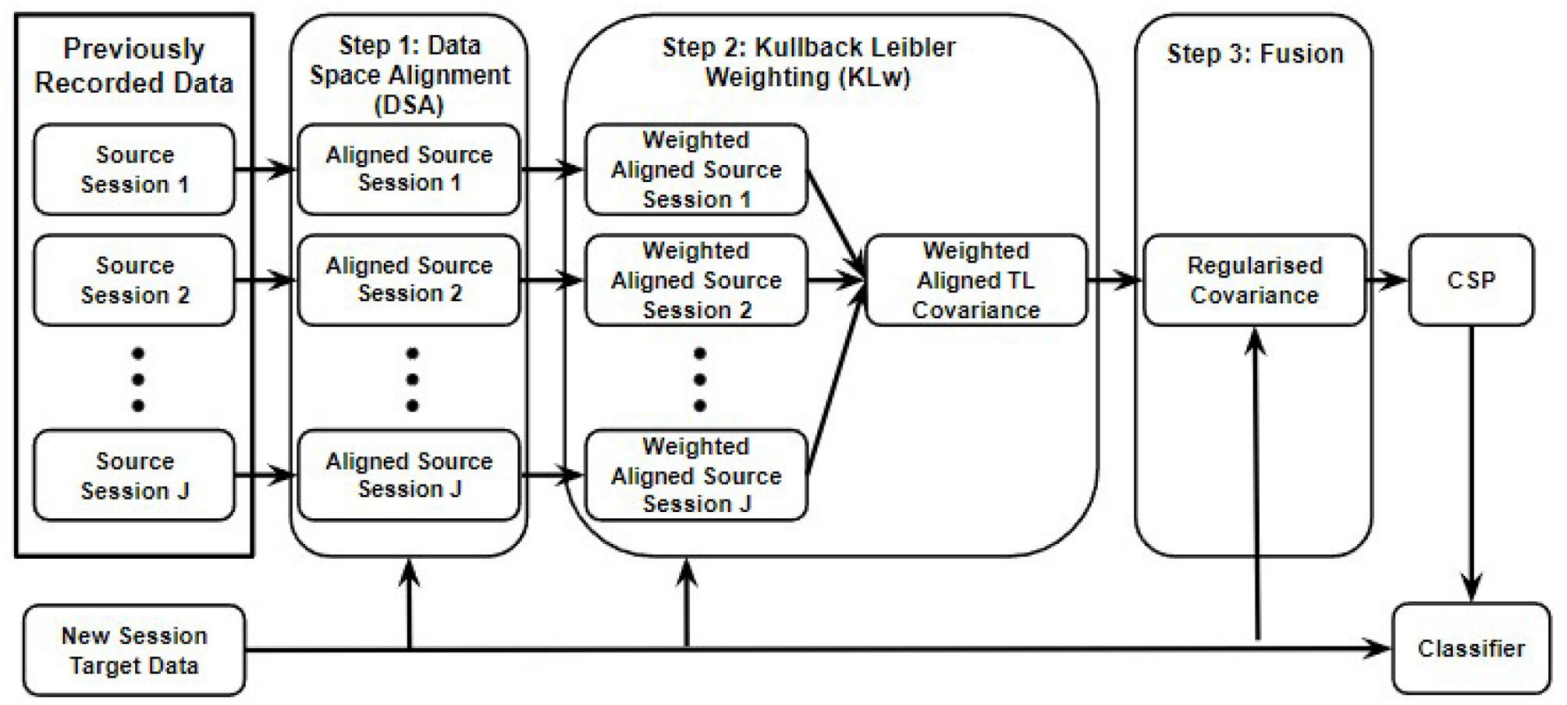
The proposed r-KLDSA algorithm is comprised of three steps, (1) the EEG data from the source sessions are aligned to the EEG data from the available target data using data space alignment, (2) weighting the aligned data of the source sessions based on their similarities with the data of the target session, (3) fusing the weighted aligned source data with the target data using a regularization method.

As can be seen in Figure 1, the proposed r-KLwDSA algorithm consists of three steps, each attempting to address one of the challenges of transfer learning in BCI. Step 1 reduces the non-stationarity between the EEG data from the source sessions and those from the target session. For this purpose, a linear transform is performed on the EEG data of each source session to reduce their distribution difference from the target data. Subsequently, step 2 defines the similarity between the EEG distributions of each linearly aligned source session and the target session using a proposed weighting mechanism. Finally, step 3 fuses the weighted aligned trials from the source sessions with the few available trials of the target session using a proposed regularization method. In fact, the regularization controls a trade-off between the target model from the new session and the weighted aligned source model from the past sessions. These three steps are explained in detail in the following subsections.

### 2.1. Linear Alignment to Reduce Non-stationarities

When performing transfer learning, one of the key issues is the presence of non-stationarities which can cause large differences in the properties of EEG data from session to session. These differences in the data space can have a very detrimental effect on the performance of transfer learning in BCI. To address this issue, we propose the use of a linear transformation, **L**_*j*_, to reduce the mismatch between the distribution of each source session, $P\left({\hat{\text{X}}}_{j},{\hat{\text{y}}}_{j}\right)$, and the distribution of the target session, *P*(**X**, **y**). For this purpose, ${\text{L}}_{j}\subset R^{ch\times ch}$ needs to be calculated such that the distribution dissimilarity between $P\left({\text{Z}}_{j},{\hat{\text{y}}}_{j}\right)$ and *P*(**X**, **Y**) is minimized where $Z_{j}=L_{j}{\hat{X}}_{j}$.

Assuming that EEG signals have Gaussian distributions (Kullback, 1997), we used Kullback Leibler (KL) divergence for Gaussian distributions to measure the distribution dissimilarity between the target session and each source session. Given two Gaussian distributions, *N*_1_(μ_**1**_, Σ_**1**_) and *N*_2_(μ_**2**_, Σ_**2**_) with μ_**1**_ and μ_**2**_ as the means and Σ_**1**_ and Σ_**2**_ as the covariance matrices, the KL divergence between *N*_1_ and *N*_2_ is measured as


(1) $$\begin{array}{l} KL[N_{1}∥N_{2}]=\frac{1}{2}(\text{tr}(\Sigma_{2}{}^{†}\Sigma_{1})+{\left(\mu_{2}-\mu_{1}\right)}^{T}\Sigma_{2}{}^{†}(\mu_{2}-\mu_{1})\\ \;-\ln(\frac{\det\Sigma_{1}}{\det\Sigma_{2}})-k), \end{array}$$

where tr, det and ln denote the trace function, the determinant function and the natural logarithm function, respectively. † and T denote the pseudo-inverse and the transpose functions, respectively. Finally, *k* refers to the dimension of the data.

As the EEG data of the source and target sessions are band-pass filtered, they have zero means. The co-variance matrices, representing the distributions of the target session, *S*, and the *j*^*th*^ source sessions, $\hat{{\text{S}}_{\text{j}}}$, are calculated using (2) and (3), respectively,


(2) $${\bar{\Sigma }}^{c}=\frac{1}{m^{c}}\sum_{i=1}^{m^{c}}\frac{X^{c,i}(X^{c,i})\text{T}}{\text{tr}(X^{\text{c},\text{i}}(X^{\text{c},\text{iT}}))},$$

(3) $${\hat{\Sigma }}_{j}^{c}=\frac{1}{{\hat{m}}_{j}^{c}}\sum_{i=1}^{{\hat{m}}_{j}^{c}}\frac{{\hat{X}}_{j}^{c,i}({\hat{X}}_{j}^{c,i})\text{T}}{\text{tr}({\hat{X}}_{j}^{c,i}({\hat{X}}_{j}^{c,i})\text{T})},$$

where, *c* denotes the class, and *m*^*c*^ is the total number of trials for the class *c*. Subsequently, the linearly transformed $\hat{{\text{S}}_{\text{j}}}$, presented as $L_{j}\hat{{\text{S}}_{\text{j}}}$, has a zero mean and the covariance matrix calculated as $L_{j}{\hat{\Sigma }}_{j}^{c}L_{j}\text{T}$. Given (1), the distribution dissimilarity between the linearly transformed *j*^*th*^ source session and the target session can be calculated as:


(4) $$KL[L_{j}\hat{S_{j}}∥S]=\frac{1}{2}\sum_{c=1}^{2}[tr({\bar{\Sigma }}^{c†}L_{j}{\hat{\Sigma }}_{j}^{c}L_{j}{}^{T})-\ln(\frac{\left(\det(L_{j}{\hat{\Sigma }}_{j}^{c}L_{j}T\right)}{\det({\bar{\Sigma }}^{c})})-ch].$$

The linear transform **L**_*j*_ aims to minimize the distribution dissimilarity between $\hat{{\text{S}}_{\text{j}}}$ and S. To calculate **L**_*j*_, the first order derivation of the loss function (5) with respect to **L**_*j*_ is computed and set to zero, as shown in (6) and (7). For more details on how optimum **L**_*j*_ has been calculated, please see the Supplementary Materials.


(5) $$A(L_{j})=\underset{L_{j}}{min}\sum_{c=1}^{2}\frac{1}{2}[\text{tr}({\bar{\Sigma }}^{c†}L_{j}{\hat{\Sigma }}_{j}^{c}L_{j}{}^{\text{T}})-\ln(\frac{\det(L_{j}{\hat{\Sigma }}_{j}^{c}L_{j}{}^{\text{T}})}{\det({\bar{\Sigma }}^{c})})-ch].$$

(6) $$\frac{dA}{dL_{j}}=\sum_{c=1}^{2}\frac{1}{2}[\frac{d}{dL_{j}}\text{tr}({\bar{\Sigma }}^{c†}L_{j}{\hat{\Sigma }}_{j}^{c}L_{j}{}^{\text{T}})-\frac{d}{dL_{j}}\ln(\det(L_{j}{\hat{\Sigma }}_{j}^{c}L_{j}{}^{\text{T}}))]=0.$$

(7) $$L_{j}=\sqrt{2}\sum_{c=1}^{2}{\left({\hat{\Sigma }}_{j}^{c}{\bar{\Sigma }}^{c†}\right)}^{†0.5}.$$

By applying the linear transform **L**_*j*_ to each of the source sessions, the KL divergence between the target and the aligned source session is minimized. This reduces the effect of the non-stationarities from session to session.

### 2.2. Weighting According to EEG Distribution Similarity

Although reducing the non-stationarity can help improve transfer learning, some source sessions can still be detrimental to the BCI. The second step of the proposed r-KLwDSA algorithm, shown in Figure 1, weights the aligned source data of each previous session to reduce the impact of adverse data while placing more weight on data that is similar to the target session. In (4), we proposed using the KL divergence between Gaussian distributions to measure dissimilarity between the aligned *j*^*th*^ source session and the target session. Subsequently, the assigned weight for the aligned *j*^*th*^ source session, ω_*j*_, presenting its distribution similarity to the target session, is calculated through equation (8),


(8) $$\omega_{j}=\frac{{\left(KL[L_{j}\hat{{\text{S}}_{\text{j}}}∥\text{S}]\right)}^{-1}}{\sum_{i=1}^{j}{\left(KL[L_{i}\hat{{\text{S}}_{\text{j}}}∥\text{S}]\right)}^{-1}},$$

where, $KL[L_{j}\hat{{\text{S}}_{\text{j}}}∥\text{S}]$ is calculated using (4). According to (8), source sessions with similar data to the data of the target session are assigned larger weights, whereas aligned source sessions with less similarity to the target session are given small weights. Consequently, the weighted aligned source data are used to calculate the co-variance matrix of past data, called the transfer learning co-variance matrix, ${\hat{\Sigma }}_{TL}^{c}$, as


(9) $${\hat{\Sigma }}_{TL}^{c}=\sum_{j=1}^{J}\omega_{j}L_{j}{\hat{\Sigma }}_{j}^{c}L_{j}^{T}.$$

### 2.3. Regularized Transfer Learning Between Past and Present Data

Transfer learning can be very effective for some of the target sessions, while for some other target sessions, the source data might be detrimental, even after weighting and alignment. These target sessions usually tend to be able to achieve high classification accuracy even when only a few target trials are available for training. As such the third step of the proposed r-KLwDSA algorithm uses a regularization method to find a trade-off between data from the previous sessions and the new data from the new target session in the final BCI model. Thus, the final regularized co-variance matrices are calculated using (10) with a regularization parameter, *r* ∈ {0, 0.1, …, 1}. The individualized regularization parameter is calculated for each target session and selected through leave-one-out cross validation method on the available target trials. The parameter achieving the highest average leave-one-out classification accuracy was then used to produce the final co-variance matrix, $\Sigma_{F}^{c}$ for class *c*. The final co-variance matrices are then used for training the Common Spatial Patterns (CSP) features (Blankertz et al., 2008), as further elaborated in Section 3.2.


(10) $$\Sigma_{F}^{c}=r{\bar{\Sigma }}^{c}+(1-r){\hat{\Sigma }}_{TL}^{c}$$

## 3. Experiment

### 3.1. Dataset

The dataset used to evaluate the proposed algorithm is known as the nBetter dataset (Foong et al., 2020). This dataset was collected by the Institute for Infocomm Research, A*STAR, Singapore to evaluate the efficacy of the Neurostyle Brain Exercise Therapy Toward Enhanced Recovery (nBETTER) system in post-stroke upper limb rehabilitation. The clinical trial obtained ethical approval from the Institution's Domain Specific Review Board (IRB), National Healthcare Group, Singapore and is registered in ClinicalTrials.gov under NCT02765334. The use of this dataset to evaluate our proposed algorithm was approved through IRB Reference: 2020-103.

All participants in the study had their first-ever stroke 3–24 months before participating the clinical trial, affecting their upper limb movements. They all provided informed consent before enrollment in the study. Potential participants attended a 40 min BCI screening session, and only those who achieved BCI accuracy above 57.5% 10-fold cross validation accuracy were invited to attend the nBetter intervention. The EEG data was collected from 24 EEG channels, placed in the international 10-20 system positioning: F3, F4, FC3, FC4, C3, C4, CP3, CP4, P3, P4, FT7, FT8, T3, T4, TP7, TP8, Fz, Oz, FCz, Cz, CPz, Pz, A1, and A2, and digitally sampled at 256 Hz for a voltage range of ±300 mV.

**Figure 2:**
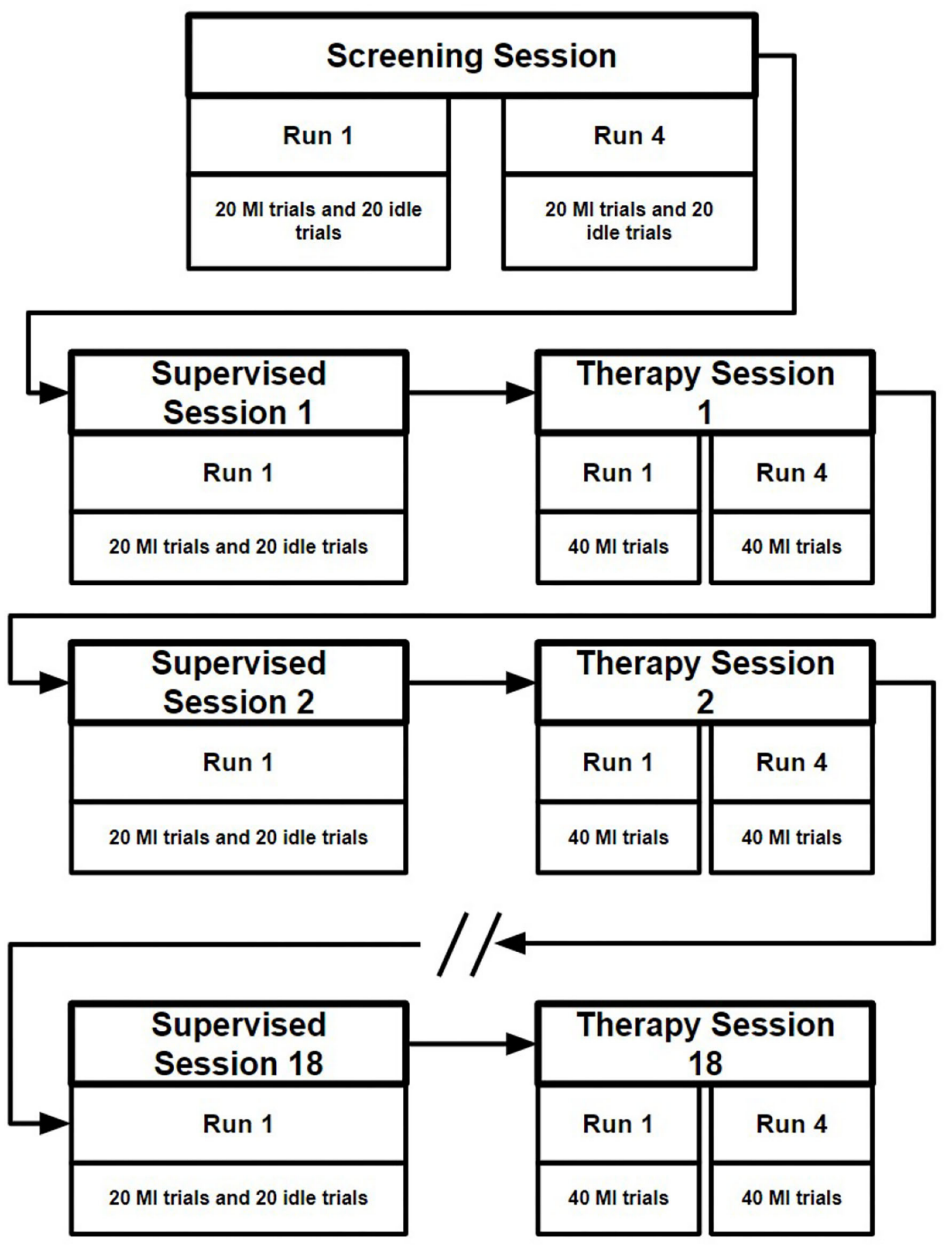
Illustration of the data collection of the nBetter dataset. First a screening session is collected at the start of the 6 weeks. Following the screening session, a supervised session followed by a therapy session is collected three times a week.

**Figure 3:**
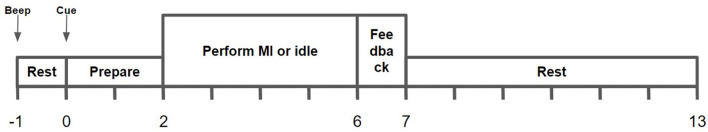
A trial from the nBetter supervised session. Each supervised session consists of 20 motor imagery (MI) trials and 20 idle trials.

In total the nBetter dataset contains the EEG data from 11 stroke patients completing one screening session, 18 supervised sessions and 18 therapy sessions supervised by an occupational therapist. The screening session, collected at the start of the study, contains four runs, each consisting of 20 idle trials and 20 motor imagery trials. For the idle class the participant was instructed to relax, whereas for the motor imagery class the participant was instructed to imagine movement of their affected hand. As shown in Figure 2, each supervised session followed by one therapy session on the same day, conducted thrice weekly over a 6 week period. In each of the supervised sessions 40 labeled trials were collected, half motor imagery and the other half idle trials. The therapy sessions contain four runs each consisting of 40 motor imagery trials. Each of the trials in these sessions lasted 13 s, as illustrated in Figure 3 with the instruction to perform motor imagery being presented for 4 s after giving the participant 2 s to prepare.

To evaluate the proposed r-KLwDSA algorithm, only the screening and supervised sessions were used. These sessions contained clearly labeled trials with equal numbers of each of the two classes. When considering each supervised session as the target session the first 10 trials of each class were used as training data, while the rest were kept for evaluation. When the supervised session was used as a source session all trials were used for transfer learning. To simulate a real world scenario the supervised sessions were evaluated chronologically. As a result, when the supervised session one was evaluated as the target session, only the screening session was used as source data. Similarly, when the supervised session 18 was used as the target session, the supervised sessions 1–17 and the screening session were used as the source sessions.

### 3.2. Data Processing

Any of the trials missing time samples were removed, other than this no artifact rejection algorithms used. A zero phase elliptic band pass filter from 8 to 35 Hz was used to filter the EEG data as this range contains the key range of frequencies that are linked to motor imagery. The band-passed EEG signals from 2.5 to 5 s after the presentation the cue were used for feature extraction. This time interval considers sufficient time for the participant to react to the motor imagery instruction. Six different feature extraction algorithms, including the proposed r-KLwDSA algorithm, are used in this article. These six algorithms utilize CSP filters to calculate the features. The CSP diagonalizes the covariance calculated for each class to find the subspace that maximizes the variance of one class while minimizing the variance of the second classes. The first and last two rows of the CSP were selected as the most discriminative spatial filters for feature extraction. The normalized variances of the spatially filtered EEG signals from the training part of the target session were used as the features to train a Linear Discrete Analysis (LDA) classifier.

Despite all using CSP for calculating the features, the covariance matrix of each class, used to calculate CSP, was obtained differently in the six applied methods. For the proposed r-KLwDSA algorithm, the covariance of each class was calculated as described above in (10). The proposed KLwDSA algorithm is a special case of the proposed r-KLwDSA algorithm with *r* = 0. In other words, the proposed KLwDSA algorithm uses the covariance matrices calculated in (9) to obtain the CSP filters. The standard session-specific (SS) algorithm is also a special case of the proposed r-KLwDSA algorithm with *r* = 1. As when *r* = 1 no transfer learning occurs making it the same as a standard CSP-LDA BCI. Thus, the SS uses only the training data available in the target session to calculate the CSP filters. The naive transfer learning algorithm, nTL, concatenates all the source sessions with equal weights and without alignments to calculate the covariance of each class for the CSP algorithm. The DSA and KLw algorithms are extensions to nTL. The DSA algorithm applies the DSA linear transform to each of the source sessions before calculating the CSP covariance matrices by concatenating the aligned source trials. The KLw algorithm weights each of the source sessions using the weighting method proposed in step 2 of the proposed algorithm without any alignment. Then the weighted covariance matrices of the source sessions are used for calculating the CSP filters. All these algorithms are compared in terms of the classification results to understand their merits and disadvantages.

## 4. Results and Discussion

### 4.1. Comparison of Classification Accuracy Results

**Figure 4:**
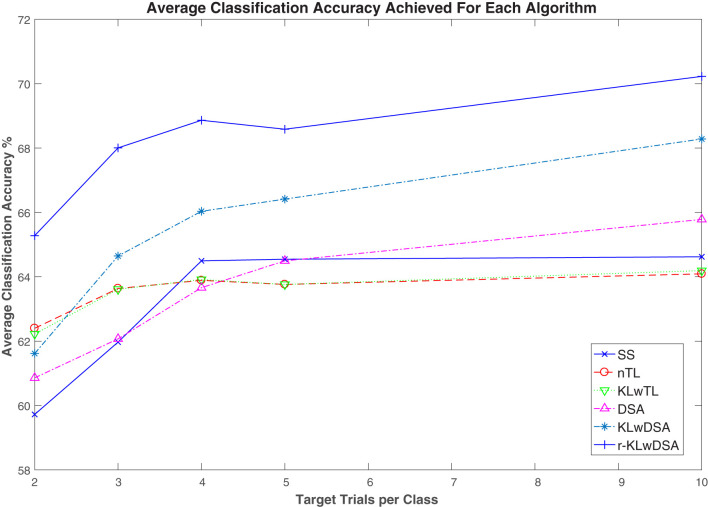
Average classification accuracy of six different algorithms across all subjects and sessions, when different number of target trials were available for calibration. SS denotes the target session-specific algorithm; nTL, naive transfer learning; proposed KLw, Kullback Liebler weighted transfer learning; proposed DSA, data space alignment transfer learning; proposed KLwDSA, aligned and weighted transfer learning; and proposed r-KLwDSA, the regularized, aligned, and weighted transfer learning algorithm.

Figure 4 shows the average classification accuracy of the six above-mentioned algorithms across all the subjects and sessions when a different number of target trials were available for BCI calibration. As shown in Figure 4, the proposed r-KLwDSA algorithm outperformed all the other algorithms across different numbers of available target trials. Given the number of available target trials between 2 to 10 per class, r-KLwDSA consistently outperformed SS by an average more than 4%. The sensitivity and specificity were also calculated for the proposed r-KLwDSA algorithm and have been included in a table in the Supplementary Materials.

A 6 (algorithms = SS, nTL, KL, DSA, KLwDSA, and r-KLwDSA) × 5 (target trials per class = 2, 3, 4, 5, and 10) × 18 (available source sessions= 1, 2, …, 18) repeated measures ANOVA test was performed on the classification results using the SPSS software. The statistical results showed that only the number of trials satisfied Mauchly Sphericity, so the Greenhouse Geisser was used to evaluate the effects of the algorithms, the number of target trials and sessions on the classification results. The results showed that the number of target trials and the algorithms had statistically significant effects on the classification accuracy with *P*-values of less than 0.001 and 0.048, respectively. The *post hoc* analysis showed using 3, 4, 5, and 10 target trials per class led to significantly better classification results compared to when we used only 2 target trials per class (*p* < 0.001). Similarly using 10 target trials per class significantly outperformed the results of using 3 trials per class (*p* = 0.008). When comparing the algorithms separately, the *post hoc* analysis showed that the proposed r-KLwDSA algorithm significantly outperformed all the other algorithms. *P*-values of less than 0.001 were obtained when comparing the proposed r-KLwDSA with the SS, nTL, DSA, KLw, and KLwDSA algorithms. The *post hoc* analysis did not show any significant difference between the SS, nTL, KLw, and DSA algorithms. Interestingly, by combining KLw and DSA, the proposed KLwDSA algorithm significantly outperformed the SS, nTL, DSA, and KLw algorithms, with the *P*-values of 0.006, 0.013, 0.012, and 0.032, respectively. We corrected the *p*-values for the multiple comparisons using the Bonferroni correction method.

**Figure 5:**
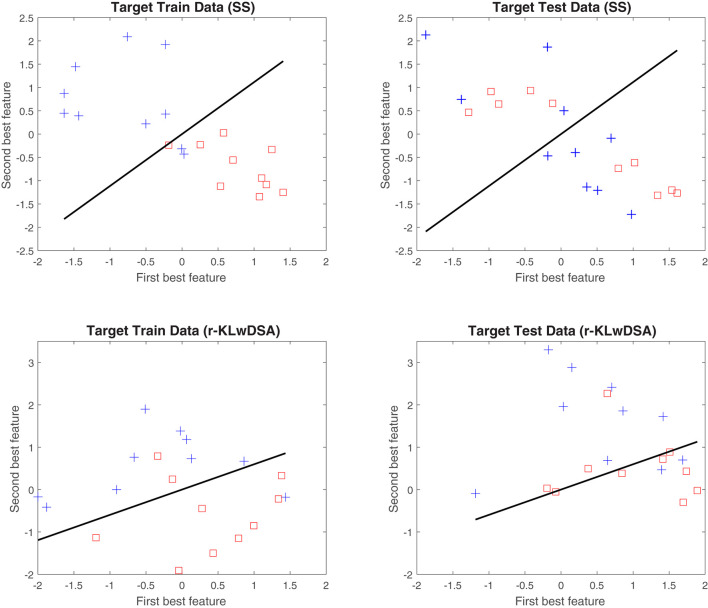
An example of the distribution of the two best features obtained by the session-specific CSP and the proposed r-KLwDSA. These features were collected from subject 6, session 16. The blue crosses and red squares denote the normalized features of the hand motor imagery and the rest class, respectively. The black line represents the LDA hyperplane obtained by the target train data.

To better understand the merits of the proposed r-KLwDSA over the standard SS algorithm, the best two features of these two algorithms, obtained using the target data from subject 6, session 16, were compared in Figure 5. Figure 5 highlights the benefit of implementing the proposed algorithm when there are only a limited number of target trials available. As shown the SS algorithm suffers greatly from overfitting due to the lack of target training trials. The SS algorithm extracts session-specific CSP features which perform very well with the available training data with only two features on the incorrect side of the hyperplane. However when transferring to the target test data the features from both classes overlap. The r-KLwDSA algorithm is not affected by this overfitting due to the integration of the source sessions data. While the trained target features overlap slightly more than the SS algorithm the test target features are much more distinctive.

### 4.2. Effects of Number of Target Trials and Source Sessions on the Performance of r-KLwDSA

Further statistical analyses were carried out to investigate the effects of the number of target trials and source sessions on the performance of the proposed r-KLwDSA algorithm. A 5 (target trials per class = 2, 3, 4, 5, and 10) × 18 (available source sessions= 1, 2,…, 18) repeated measures ANOVA test was performed on the r-KLwDSA classification results. Mauchly Sphericity was satisfied for the number of trials per class, so the sphericity assumed results were used. The ANOVA results confirmed that the number of available target trials had a main affect on the classification accuracy with a *P*-value of less than 0.001. This is aligned with previous literature, as increasing the number of target trials improves the estimation of the average target trials for each class. The improved average target trial results in a better DSA alignment and a more accurate KL weighting, consequently improving the r-KLwDSA accuracy.

While increasing the available target trials significantly improved the classification accuracy, the number of available source sessions did not have a main effect on the classification results of the proposed r-KLwDSA algorithm (*P* = 0.472). A potential factor contributing to the lack of a significant effect of the number of source sessions on the r-KLwDSA results could be the non-stationarity of the EEG signals. The users' EEG signals vary from session to session, and these variations can be significant over extended periods. Thus, increasing the number of the source data could not necessarily improve the BCI accuracy. Please note that to mimic practical scenarios, we considered the data chronologically and used all the available source sessions for training r-KLwDSA. Thus, our results did not make a direct comparison between the different number of source sessions as by increasing the number of the source sessions the target sessions were changed. To better analyse the impact of number of source sessions on the r-KLwDSA performance, we fixed the target session to session 18 and used different numbers of the nearest source sessions. However still we did not observe a statistically significant effect of number of source sessions on rKLwDSA results. The details of these results are available in the Supplementary Material.

### 4.3. Change in Classification Accuracy for Those Encountering BCI Deficiency

**Figure 6:**
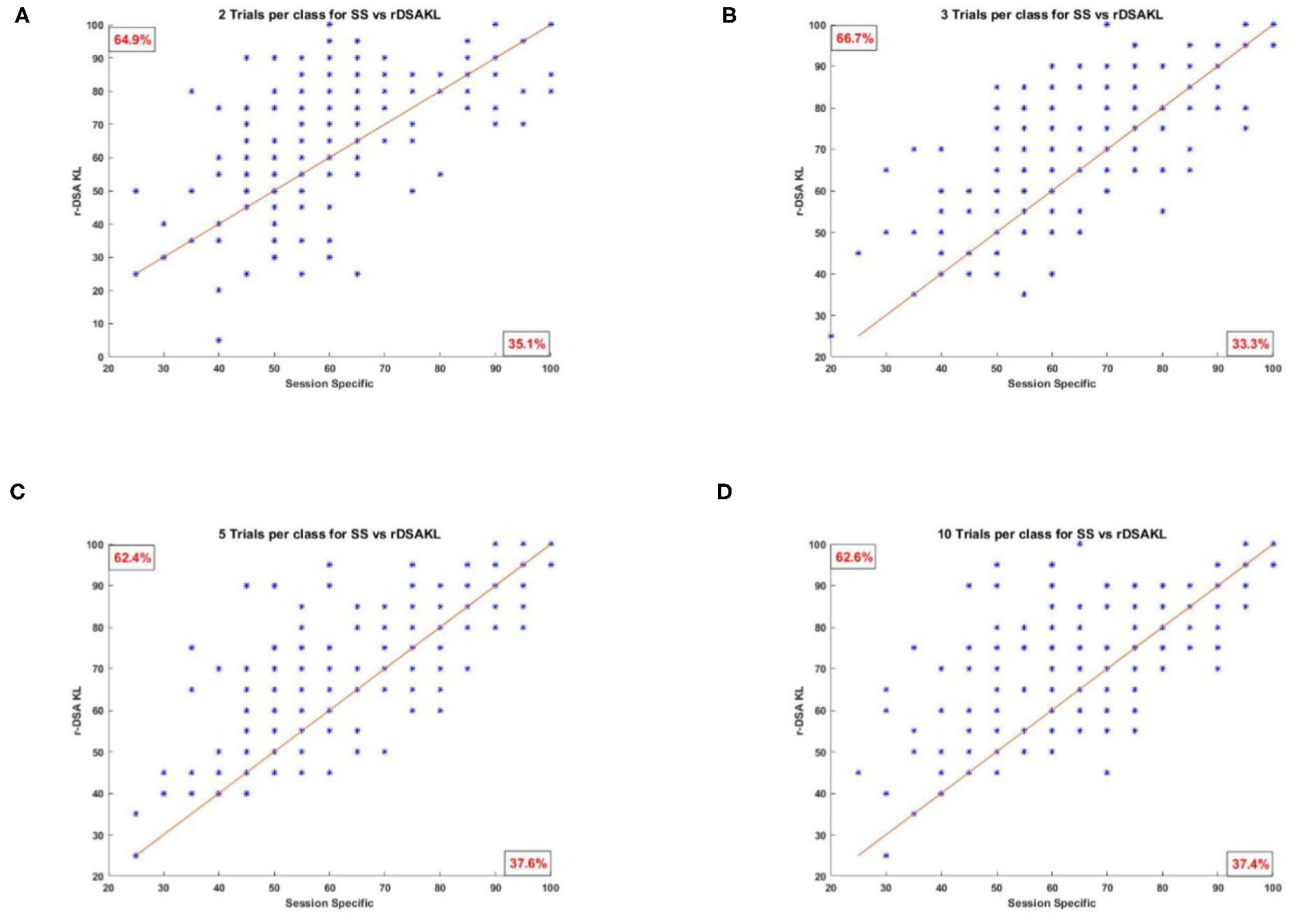
Four scatter plots showing the SS classification accuracy against the classification accuracy of the proposed r-KLwDSA algorithm. Each star represents one test session of a patient. **(A)** Two trials per class, 64.9% users improve with r-KLwDSA. **(B)** Three trials per class, 66.7% users improve with r-KLwDSA. **(C)** Five trials per class, 62.4% users improve with r-KLwDSA. **(D)** Ten trials per class, 62.6% users improve with r-KLwDSA.

Figure 6 presents scatter plots showing all the classification results obtained using SS against those obtained using the proposed r-KLwDSA algorithm, when 2, 3, 5, and 10 target trails were available for BCI calibration. As can be seen, compared to the SS algorithm, the increased classification accuracy from using the proposed r-KLwDSA algorithm was pronounced for stroke users encountering BCI deficiency (i.e., SS accuracy less than 60%). As expected, increasing the number of available target trials led to a larger improvement in the classification accuracy of the users who were identified as BCI deficient using the SS algorithm.

To better investigate the benefit of using r-KLwDSA, Table 1 splits the 198 available target sessions based on the classification accuracy achieved by the SS algorithm, when 10 target trials per class were available for BCI calibration. Impressively, for the total 73 sessions where the SS encountered BCI deficiency (i.e., accuracy below 60%), the use of r-KLwDSA yielded a significant increase in the classification accuracy with an average improvement of 13.22% and *p*-value of 0. Moreover, the proposed r-KLwDSA significantly improved the classification accuracy of the total 87 sessions achieving between 60% and 85% accuracy using the SS algorithm. However, the observed average improvement was smaller, with the average accuracy increasing by 2.99%. On the contrary, sessions with a SS classification accuracy more than 85% observed an average decrease in the accuracy when r-KLwDSA was applied. This shows when the session-specific model performs very well, adding source sessions to the model could be detrimental and the proposed regularization method could not deal with it as expected. This could be because the regularization values were chosen using cross validation on only very few target trials, which increases the risk of over fitting. Thus, there is a need to investigate novel ways to find the optimum regularization values, particularly for those with very good initial SS performance.

**Table 1:** The sessions are separated into those achieving below 60%, between 60 and 85% and above 85% classification accuracy using the session specific (SS) BCI model, when there were 10 target trials per class available for calibration.

	**Below 60%**	**60–85%**	**85–100%**
SS Mean Acc	45.82%	68.56%	91.71%
r-KLwDSA Mean Acc	59.04%	71.55%	88.68%
SS Count	73	87	38
*P*-value	0	0.016	0.003

*The average classification accuracy achieved by these sessions using the proposed r-KLwDSA and the session specific BCI are presented with the p-value calculated from the t-test between them*.

Considering the r-KLwDSA results and regardless of the number of target trials available, we observed consistent improvements in the classification accuracy of the sessions with BCI deficient SS models. For those sessions, the proposed r-KLwDSA algorithm improved the classification accuracy by an average of 9.29, 9.54, 8.93, 9, and 13.22% for 2, 3, 4, 5, and 10 trials per class, respectively. Importantly, the observed improvements in the classification accuracy were significant for all these different number of available target trials with *P*-values of less than 0.001.

In summary, Figure 6 and Table 1 show that the proposed r-KLwDSA could potentially reduce the number of sessions encountering BCI deficiency while limiting the calibration time to less than 4 min. Thus, r-KLwDSA could help more stroke patients have a meaningful and potentially effective BCI-based rehabilitation.

### 4.4. Impact of Number of Source Sessions on the Regularization Value

**Figure 7:**
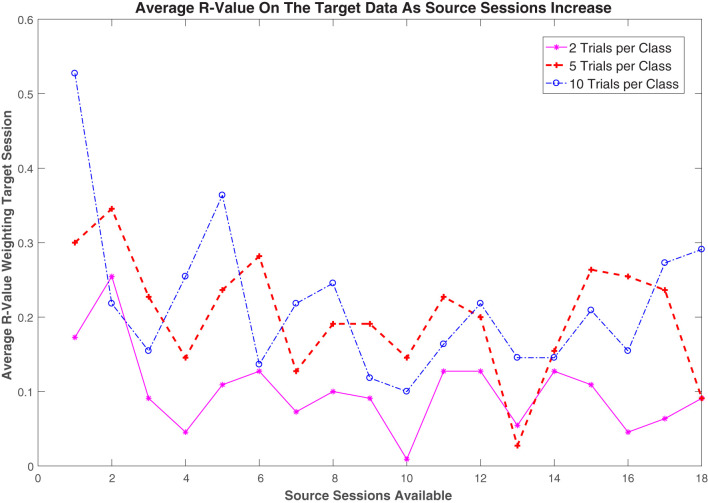
The average *r*-value used for regularizing the proposed r-KLwDSA algorithm for each of the target trials per class is plotted against the number of source sessions available. In the proposed r-KLwDSA, the *r*-value, *r*, is used to weight the available target trials while 1−*r* is used to weight the aligned weighted source trials.

Figure 7 illustrates the effects of the number of available target trials and source sessions on the regularization value in the proposed r-KLwDSA algorithm. The regularization value, *r*, defines a trade-off between the weighted aligned source trials and the target trials in the final r-KLwDSA model. Figure 7 shows larger weights were given to the target trials when more target trials per class were available for training the r-KLwDSA model. For example, given one source session available, when 10 target trials per class were used for training, the target trials on average got weighted as *r* = 0.52, whereas the average obtained *r* was 0.17 when there were two target trials per class available for calibration. These results suggest that when more target trials are getting available for calibration, r-KLwDSA gets more similar to the target session-specific model rather than the transfer learning model extracted from the source sessions. However, when there are only a couple of target trials available for training, although the proposed r-KLwDSA algorithm still finds them useful, the focus has to be on the source data available as a clear representation of the target session cannot be calculated from the limited data available.

When there were less than 5 source sessions available, the effects of the number of source sessions on *r* was opposite to the effect of the number of target trials. Figure 7 shows when there was only one previous source session available, the average *r*-value was high; however, as the number of sessions available increased, the average weight given to the target trials decreased. This drop in the *r*-value is presumably due to the increasing amount of source data available for transfer learning. As the amount of transfer learning data increased, the proposed r-KLwDSA could find more source sessions which were similar to the target session and could produce more robust features without relying too much on the available target trials. However, this trend is not consistent as the further increase in the number of available sessions did not lead to a further decrease in the *r*-value. This end to the trend could be due to the user adapting to using the BCI over time. As the user continues to use the BCI for rehabilitation, they would start learning how to produce more consistent and separable target EEG signals. Due to this the proposed r-KLwDSA algorithm would adapt to this change and increase the *r*-value to rely more on the target data when calibrating the BCI model.

### 4.5. Limitations and Future Work

Although the results collected show that the proposed r-KLwDSA performed best for the majority of the stroke patients in a few cases other method performed better. In particular for some users the SS performed much better for a couple of sessions.

Ideally if the correct regularization parameter *r* was calculated for each session the proposed r-KLwDSA should always outperform the standard SS algorithm. Utilizing regularization improved the classification accuracy however applying leave-one-out cross validation to select the *r* value is rather lacking. This method is prone to overfitting due to the limited number of target trials available. Finding a better alternative method to calculate the *r* value that works well with limited trials would be very beneficial.

The current proposed r-KLwDSA assumes that there are no non-stationarities within each session which is not an always correct assumption. Further work could be done to reduce the effects of these non-stationarities. Different variants of CSP have been produced to reduce the effects of these non-stationarities such as the KL-CSP and DTW-CSP (Arvaneh et al., 2013b; Azab et al., 2019a). Alternatively, online adaptation algorithms have also been developed to reduce these non-stationarities and could further improve the classification accuracy (Arvaneh et al., 2013a).

## 5. Conclusion

This article proposed a novel algorithm for transfer learning combining linear alignment, weighting and regularization to reduce the calibration time for long-term BCI users. The linear alignment aimed to reduce the non-stationarity between the source and target sessions, whereas the weighting mechanism adjusted the impact of each source session on the BCI model based on its similarity to the target data. Finally, the regularization step combined the weighted aligned source data and the few available target data to build the final BCI model. The proposed algorithm significantly outperformed the session-specific model and a number of other state-of-the -art transfer learning algorithms when the number of available target trials was very few and the number of available source sessions was between 1 to 18. Importantly, the proposed algorithm remarkably reduced the number of BCI sessions with deficient session-specific accuracy (i.e., less than 60%) with an average accuracy improvement of around 10%.

## Data Availability Statement

The data analyzed in this study is subject to the following licenses/restrictions: the dataset was collected by the Agency for Science, Technology and Research (A^*^STAR) Singapore and contains medical data. As such this dataset can only be used with IRB approval from A^*^STAR. Requests to access these datasets should be directed to KA, kkang@i2r.a-star.edu.sg.

## Author Contributions

All authors listed have made a substantial, direct, and intellectual contribution to the work and approved it for publication.

## Funding

This research was supported by the UK Medical Research Council (MRC), grant number MC_PC_19051 and the Agency for Science, Technology and Research (A^*^STAR) Singapore.

## Conflict of Interest

The authors declare that the research was conducted in the absence of any commercial or financial relationships that could be construed as a potential conflict of interest.

## Publisher's Note

All claims expressed in this article are solely those of the authors and do not necessarily represent those of their affiliated organizations, or those of the publisher, the editors and the reviewers. Any product that may be evaluated in this article, or claim that may be made by its manufacturer, is not guaranteed or endorsed by the publisher.
