# Supplementary material for: A Transfer Learning Algorithm to Reduce Brain-Computer Interface Calibration Time for Long-Term Users
Source: Front Neuroergon. 2022 Apr 21;3:837307. doi: 10.3389/fnrgo.2022.837307 (PMC10790953; doi:10.3389/fnrgo.2022.837307)
Supplement: Supplementary file 1 [file Data_Sheet_1.pdf]

# Supplementary Material

## 1 MATHEMATICAL PROOF OF EQUATION 7

To obtain the optimal $\mathbf{L}_j$, presented in (7), we need to calculate the first-order derivative of (4), with respect to $\mathbf{L}_j$, and set it to zero. To do this we use the properties presented below [28]:

$$\mathrm{tr}(\mathbf{ABC}) = \mathrm{tr}(\mathbf{CAB}) = \mathrm{tr}(\mathbf{CBA}) \tag{S1}$$

$$\frac{d}{d\mathbf{L}_j}\mathrm{tr}(\mathbf{A}) = \mathrm{tr}(\frac{d\mathbf{A}}{d\mathbf{L}_\mathrm{j}}) \tag{S2}$$

$$\frac{d}{d\mathbf{L}_j}\det(\mathbf{A}) = \det(\mathbf{A})\mathrm{tr}(\mathbf{A}^{-1}\frac{d\mathbf{A}}{d\mathbf{L}_\mathrm{j}}) \tag{S3}$$

where **A**, **B**, and **C** are real matrices. As the EEG covariance matrices are positive and symmetric we can conclude that:

$$\frac{d}{d\mathbf{L}}\mathrm{tr}(\overline{\mathbf{\Sigma}}^{\mathrm{c}\dagger}\mathbf{L_j}\hat{\mathbf{\Sigma}}^{\mathrm{c}}_{\mathrm{j}}\mathbf{L_j}^{\mathrm{T}}) = \frac{d}{d\mathbf{L}}\mathrm{tr}(\hat{\mathbf{\Sigma}}^{\mathrm{c}}_{\mathrm{j}}\mathbf{L_j}\overline{\mathbf{\Sigma}}^{\mathrm{c}\dagger}\mathbf{L_j}^{\mathrm{T}}) \tag{S4}$$

$$\frac{d}{d\mathbf{L}}\mathrm{tr}(\overline{\mathbf{\Sigma}}^{\mathrm{c}\dagger}\mathbf{L_j}\hat{\mathbf{\Sigma}}^{\mathrm{c}}_{\mathrm{j}}\mathbf{L_j}^{\mathrm{T}}) = 2\mathrm{tr}(\overline{\mathbf{\Sigma}}^{\mathrm{c}\dagger}\mathbf{L_j}\hat{\mathbf{\Sigma}}^{\mathrm{c}}_{\mathrm{j}}) = 2\mathrm{tr}(\hat{\mathbf{\Sigma}}^{\mathrm{c}}_{\mathrm{j}}\overline{\mathbf{\Sigma}}^{\mathrm{c}\dagger}\mathbf{L_j}) \tag{S5}$$

$$\frac{d}{d\mathbf{L}_j}\ln(\det(\mathbf{L_j}\hat{\mathbf{\Sigma}}^{\mathbf{c}}_{\mathbf{j}}\mathbf{L_j}^{\mathrm{T}})) = \frac{\det(\mathbf{L_j}\hat{\mathbf{\Sigma}}^{\mathbf{c}}_{\mathbf{j}}\mathbf{L_j}^{\mathrm{T}})}{\det(\mathbf{L_j}\hat{\mathbf{\Sigma}}^{\mathbf{c}}_{\mathbf{j}}\mathbf{L_j}^{\mathrm{T}})}2\mathrm{tr}(\hat{\mathbf{\Sigma}}^{\mathbf{c}}_{\mathbf{j}}\mathbf{L_j}^{\mathrm{T}}(\mathbf{L_j}\hat{\mathbf{\Sigma}}^{\mathbf{c}}_{\mathbf{j}}\mathbf{L_j}^{\mathrm{T}})^{-1}) \tag{S6}$$

$$\frac{d}{d\mathbf{L}_j}\ln(\det(\mathbf{L_j}\hat{\mathbf{\Sigma}}^{\mathbf{c}}_{\mathbf{j}}\mathbf{L_j}^{\mathrm{T}})) = \frac{d}{d\mathbf{L}_j}\ln(\det(\mathbf{L_j}\hat{\mathbf{\Sigma}}^{\mathbf{c}}_{\mathbf{j}}\mathbf{L_j}^{\mathrm{T}})) = 2\mathrm{tr}(\mathbf{L_j}^{-1}) \tag{S7}$$

By substituting equation (S5) and (S7) into equation (6), we find

$$\frac{dA}{d\mathbf{L}_j} = \sum_{c=1}^{2}\mathrm{tr}(\hat{\mathbf{\Sigma}}^{\mathrm{c}}_{\mathrm{j}}\overline{\mathbf{\Sigma}}^{\mathrm{c}\dagger}\mathbf{L_j} - \mathbf{L_j}^{-1}) = 0 \tag{S8}$$

One of the solutions for equation (S8) is

$$\hat{\mathbf{\Sigma}}_j^1 \overline{\Sigma}^{1\dagger}\mathbf{L_j} + \hat{\mathbf{\Sigma}}_j^2 \overline{\mathbf{\Sigma}}^{2\dagger}\mathbf{L_j} - 2\mathbf{L_j}^{-1} = 0 \tag{S9}$$

This solution can then be simply re-arranged as equation (S9).

## 2 EFFECTS OF INCREASING SOURCE SESSION AVAILABILITY ON ACCURACY

One of the interesting findings of this paper is how increasing the available source session does not always lead to an improvement in the classification accuracy. Figure S1 shows the changes in the classification accuracy for target session 18 for all 11 subjects. First only the previous session, session 17, is used to train the BCI then session 17 and 16 are used followed by sessions 17, 16 and 15 and so on until all the previous data has been used.

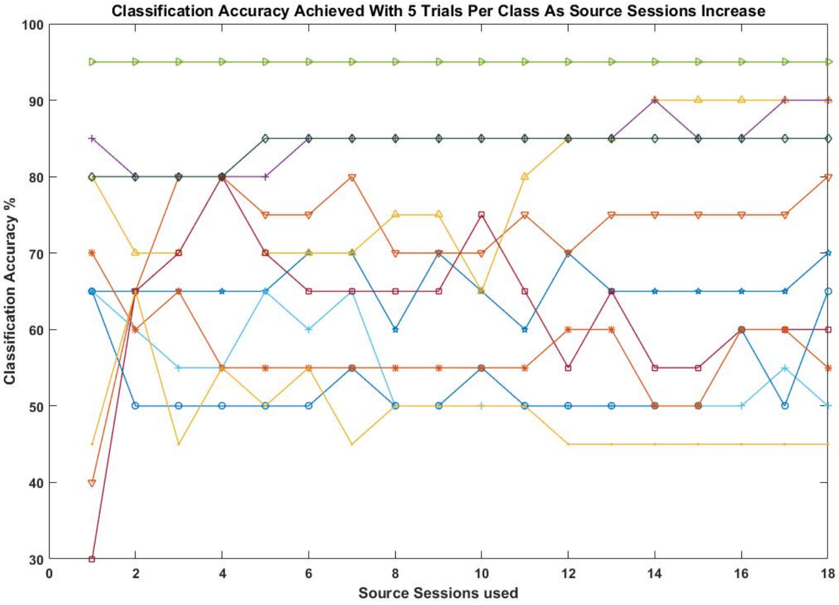


Figure S1: Classification results of the proposed r-KLwDSA algorithm using 5 trials per class from the session 18 as the target session and different number of source sessions. Each curve presents one participant.

Figure S2 shows the classification accuracy across all 11 subjects as a box plot. The highest mean classification accuracy across all the users is when there are 4 source sessions available. However the actual best number of source sessions varies significantly from user to user.

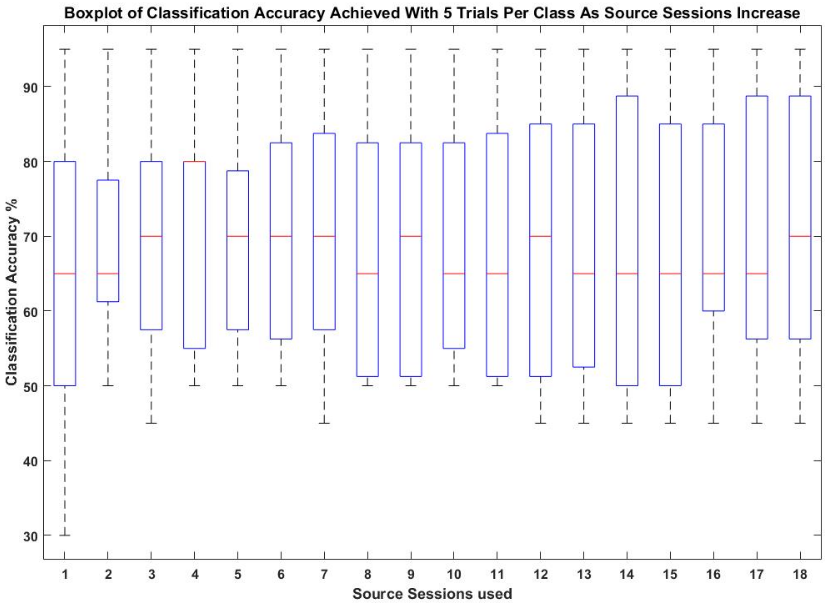


Figure S2: Box-plots of the classification results for the proposed r-KLwDSA algorithm using 5 trials per class from the session 18 as the target session and different number of source sessions.

## 3 AVERAGE SENSITIVITY AND SPECIFICITY OF EACH ALGORITHM

Table 1 compares the average Sensitivity and specificity of the proposed r-KLwDSA algorithm with the Sensitivity and specificity of SS, nTL, KL, DSA, and KLwDSA.

| | **2 target trials per class** | | | **3 target trials per class** | | | **4 target trials per class** | | | **5 target trials per class** | | | **10 target trials per class** | | |
|---|---|---|---|---|---|---|---|---|---|---|---|---|---|---|---|
| | Mean Accuracy | Mean Specificity | Mean Sensitivity | Mean Accuracy | Mean Spherecity | Mean Sensitivity | Mean Accuracy | Mean Specificity | Mean Sensitivity | Mean Accuracy | Mean Specificity | Mean Sensitivity | Mean Accuracy | Mean Specificity | Mean Sensitivity |
| **SS** | 59.72 | 60.20 | 59.24 | 61.97 | 61.62 | 62.32 | 64.49 | 62.83 | 66.16 | 64.55 | 63.79 | 65.30 | 64.62 | 65.51 | 63.74 |
| **nTL** | 62.40 | 59.14 | 65.66 | 63.64 | 62.02 | 65.25 | 63.89 | 62.42 | 65.35 | 63.76 | 61.67 | 65.86 | 64.09 | 59.09 | 69.09 |
| **KL** | 62.22 | 58.89 | 65.56 | 63.61 | 61.97 | 65.25 | 63.91 | 62.42 | 65.40 | 63.76 | 61.67 | 65.86 | 64.19 | 59.65 | 68.74 |
| DSA | 60.86 | 59.65 | 62.07 | 62.07 | 62.63 | 61.52 | 63.66 | 63.08 | 64.24 | 64.49 | 64.34 | 64.65 | 65.78 | 61.31 | 70.25 |
| KLwDSA | 61.62 | 58.79 | 64.44 | 64.65 | 62.32 | 66.97 | 66.04 | 63.43 | 68.64 | 66.41 | 64.14 | 68.69 | 68.28 | 64.80 | 71.77 |
| r-KLwDSA | 65.28 | 62.63 | 67.93 | 68.01 | 65.51 | 70.51 | 68.86 | 64.55 | 73.18 | 68.59 | 64.75 | 72.42 | 70.23 | 67.27 | 73.18 |

**Table S1.** The average classification accuracy, specificity and sensitivity are shown for each algorithm as the number of target trials increases. These averages are calculated across all the users and sessions.
